# Cholera

**Published:** 1884-07-20

**Authors:** 


### *CHOLERA.*

Asiatic Cholera is now raging in France, first appearing at Toulon, then at Marseilles and other cities of France. The whole civilized world is now on the lookout to ward off the malady, if possible. The great epidemics of this scourge have usually struck the American continent at Quebec, Montreal or New York; thence to Philadelphia, Baltimore and the Southern seaport cities, and, moving westward, follows the commercial lines of travel up the Mississippi river and its tributaries, being confined to the limestone formations. Though sanitary precautions are being generally observed, it is probable that the disease will visit us, reaching us, perhaps, not before next spring or summer. Hope is everywhere entertained that improved sanitation and advances in medical treatment will greatly restrict its ravages and ameliorate its terrors.

